# Managing Viral Emerging Infectious Diseases via Current and Future Molecular Diagnostics

**DOI:** 10.3390/diagnostics13081421

**Published:** 2023-04-15

**Authors:** Mustafa Altindiş, Elmas Pınar Kahraman Kilbaş

**Affiliations:** 1 Medical Microbiology Department, Faculty of Medicine, Sakarya University, Sakarya 54050, Türkiye; maltindis@sakarya.edu.tr; 2 Medical Laboratory Techniques, Vocational School of Health Services, Fenerbahce University, Istanbul 34758, Türkiye

**Keywords:** emerging viral infections, PCR, next-generation sequencing, CRISPR-Cas, LAMP

## Abstract

Emerging viral infectious diseases have been a constant threat to global public health in recent times. In managing these diseases, molecular diagnostics has played a critical role. Molecular diagnostics involves the use of various technologies to detect the genetic material of various pathogens, including viruses, in clinical samples. One of the most commonly used molecular diagnostics technologies for detecting viruses is polymerase chain reaction (PCR). PCR amplifies specific regions of the viral genetic material in a sample, making it easier to detect and identify viruses. PCR is particularly useful for detecting viruses that are present in low concentrations in clinical samples, such as blood or saliva. Another technology that is becoming increasingly popular for viral diagnostics is next-generation sequencing (NGS). NGS can sequence the entire genome of a virus present in a clinical sample, providing a wealth of information about the virus, including its genetic makeup, virulence factors, and potential to cause an outbreak. NGS can also help identify mutations and discover new pathogens that could affect the efficacy of antiviral drugs and vaccines. In addition to PCR and NGS, there are other molecular diagnostics technologies that are being developed to manage emerging viral infectious diseases. One of these is CRISPR-Cas, a genome editing technology that can be used to detect and cut specific regions of viral genetic material. CRISPR-Cas can be used to develop highly specific and sensitive viral diagnostic tests, as well as to develop new antiviral therapies. In conclusion, molecular diagnostics tools are critical for managing emerging viral infectious diseases. PCR and NGS are currently the most commonly used technologies for viral diagnostics, but new technologies such as CRISPR-Cas are emerging. These technologies can help identify viral outbreaks early, track the spread of viruses, and develop effective antiviral therapies and vaccines.

## 1. Introduction

Molecular diagnostic methods for viral infectious diseases have been developing rapidly in recent times. The primary aim of nucleic acid tests (NATs) is to offer rapid results in order to provide quality healthcare at an affordable price [1]. Empirical data and modeling studies have supported a better understanding of and led to improvements in the detection rate of viral diseases, causing reductions in hospitalizations and antibiotic use [2]. Polymerase chain reaction (PCR), real-time PCR (RT-PCR), nucleic acid sequence-based amplification (NASBA), etc., have been used in the laboratory diagnosis of viruses and for genotyping and quantitative testing. Amplification methods have the advantages of high sensitivity and repeatability [3,4,5]. There are many qualitative and quantitative molecular diagnostic methods based on PCR [6,7]. The NASBA method allows for the detection of an active infection by detecting the viral RNA genome and viral messenger RNA (mRNA), but RT-PCR techniques are still the most commonly used tests for diagnosing viral diseases [8]. 

Today, many molecular diagnostic methods have been replaced by automatic devices that can work with smaller liquid amounts in shorter times and provide high-sensitivity monitorable results. Automated systems have some advantages, including lower contamination risk, reduced diagnostic time, enhanced performance and speed, lower microbial load detection, and lower costs [9]. In this review, we discuss the current and emerging molecular diagnostic methods used in the treatment of viral diseases.

## 2. Current Molecular Methods for the Identification of Viral Infections

Molecular techniques for the identification of viruses are mainly nucleic acid-based tests (NATs). These are multi-step methods in which the pathogenic nucleic acid is first purified by extraction from biological samples such as blood, urine, and saliva and then analyzed by PCR. The catalytic effect of the polymerase enzyme and the amplification of specific genetic sequences of a single microorganism are carried out through thermal cycling. To date, the PCR reaction has been extensively studied and optimized for the development of RT-PCR, which allows for the quantitation of amplified genetic sequences to be detected quantitatively using a fluorescent label [10]. Although NATs are frequently used in the diagnosis of infectious diseases, due to the time, cost, and effort involved and the need for expensive machines, they have begun to be replaced by time-saving, portable, and more integrated technologies named point of care (POC). This was particularly true during the COVID-19 pandemic [11]. 

### 2.1. Real-Time Quantitative Polymerase Chain Reaction (RT-qPCR)

RT-qPCR measures the amount of target amplicon during the reaction. This measurement is simplified by DNA intercalation dyes or fluorescently labeled probes. To use these dyes, primers must be highly optimized and not generate non-specific amplicons. Fluorescently labeled probes (e.g., hydrolysis or hybridization probes) are target-specific sequences [12]. The level of fluorescence in the PCR sample is monitored and compared with the primary concentration of the target in the specimen, hence providing quick quantification.

RT-qPCR has both advantages and disadvantages regarding the development of rapid diagnostic methods in the management of viral diseases. Promising high specificity is an advantage when using probes such as hydrolysis or hybridization probes since the primers and probe must connect the target sequence to obtain the signal. In the case of time constraints, while the qPCR method gives a relatively fast result, the sample preparation required in the preparation phase can be a retarding factor. While there are several commercially available kits that offer a simpler and quicker process of isolation of DNA or RNA, they come at a higher price [13]. Consequently, in relation to the initial cost of qPCR materials, the newly available kits can present challenges for developing countries where these methods are beginning to be incorporated into routine operations.

### 2.2. Loop-Mediated Isothermal Amplification (LAMP)

This is based on automated loop strand substitution DNA synthesis, which is carried out at 60–65 °C for 45–60 min in the presence of LAMP, Bst DNA polymerase, dNTPs, specific primers, and the target DNA [14]. The LAMP method utilizes a DNA polymerase with high helical displacement activity and four specially constructed primers (internal and external) that recognize six different sequences in the target DNA. One of the inner primers initiates the LAMP reaction, while the other is used for self-priming in later steps. The LAMP amplification reaction consists of three stages: (i) production of the starting material, (ii) amplification and elongation cycle, and (iii) recycling. The target amplicon gains high specificity thanks to the recognition of six distinct sequences at the start of LAMP and the recognition of four sequences at later stages. Four primers are used simultaneously to initiate DNA synthesis from the original DNA to form a stem-loop DNA for the next LAMP cycle where the target is recognized by the four sequences. Thus, its target selectivity is expected to be greater compared to PCR [15]. 

The existing evidence has shown that different LAMP assays are on average up to 100 times more sensitive than conventional RT-PCR methods for detecting SARS-CoV-2 RNA in patient samples [16,17,18]. (Table 1). However, it also has disadvantages as it prevents the addition of an internal PCR inhibition control that requires replication during testing. The method is also complex and requires a complex primer design system that can limit the selection or specificity of target sites [19]. 

### 2.3. Fluorescent In Situ Hybridization (FISH)

FISH is a type of molecular cytogenetic technique that uses fluorescent probes that bind to chromosome fragments to provide a high degree of complementarity in a nucleotide sequence. Fluorescence microscopy is used to determine where on the chromosome the fluorescently labeled probe binds [41]. The implementation of FISH for the detection and identification of viral pathogens has advanced significantly since the turn of the 21st century [42]. The method has been applied to investigate the localization of viral nucleic acids in tissues and organs infected with viruses such as SARS-CoV-2 and HIV and to identify viruses [43,44,45,46]. (Table 1).

### 2.4. Sequencing Methods

In nucleotide sequencing methods, the nucleotide sequence in a piece of DNA is determined. The sequencing of the virus genome is most widely done by the dideoxy-chain termination method, also known as “Sanger Sequencing”. This method must contain a single-stranded DNA sequence, DNA primer, DNA polymerase, deoxynucleoside triphosphates (dNTPs), and dideoxynucleotides triphosphates (ddNTPs). The viral nucleic acid is split into four reactions, adding one of the four dideoxynucleotides (ddNTPs; ddATP/GTP/CTP/TTP). ddNTPs are used to terminate the elongation of the DNA chain. ddNTPs are similar in chemical structure to dNTPs but lack the 3’-OH group required for a phosphodiester bonding to form between the two nucleotides, which stops DNA polymerase from elongating when a ddNTP is added. The DNA of the virus to be sequenced is denatured into single strands by heat, and a primer is tied to one of the template sequences. Primers or nucleotides are labeled as radioactive or fluorescent. The key point of this technique is that all reactions start from the identical nucleotide and finish with a specific nucleotide. An automatic sequencing method has been developed to speed up the sequencing process and increase detection sensitivity. In this technique, the reactions are performed in a tube with four ddNTPs marked with different colors in the form of fluorescents of various wavelengths [47]. DNA sequencers divide nucleotide strands according to their size by capillary electrophoresis. Because the four dyes radiate at various wavelengths, the identification of the bands is read according to the wavelength at which they fluoresce. The outcomes are then presented as a chromatogram. The most widely used method for the whole-genome sequencing of RNA viruses is to design whole-genome spanning amplicons and amplify target regions by RT-PCR [48]. 

**Figure 1:**
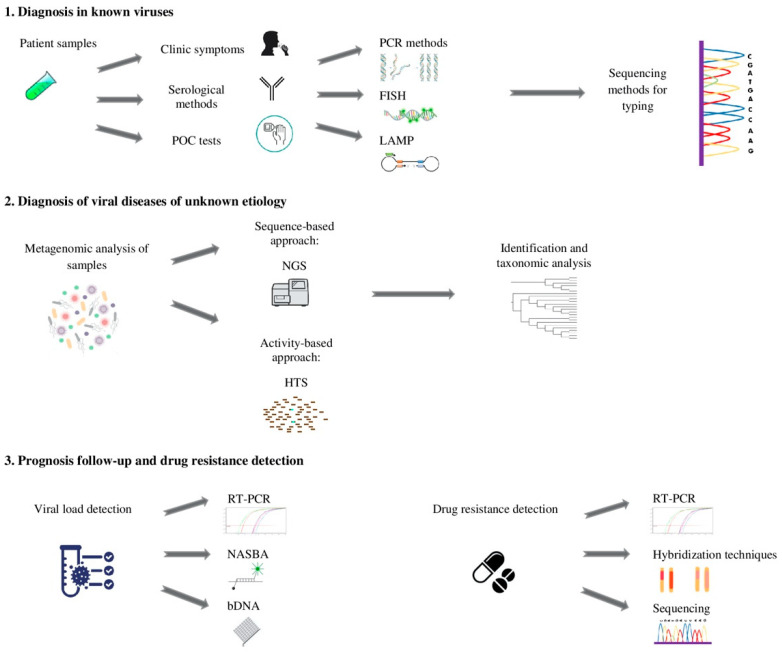
Flow chart used in the management of viral diseases: 1. An approach for the identification of known viruses; 2. The evaluation of virus populations for the detection of emerging viruses; 3. Appropriate techniques for viral disease prognosis monitoring and drug resistance.

Next-generation sequencing (NGS) methods are high-speed and efficient techniques to analyze large volumes of nucleotide sequences at once. The major advantages of NGS platforms are the ability to both detect the nucleotide sequence from single DNA fragments of a large sequence and eliminate the need to clone vectors before sequence detection. NGS platforms can be applied in different areas in many clinical and research laboratories for reasons such as reducing costs, enabling the diagnosis of viral infectious diseases and molecular epidemiology of etiological pathogens, being compatible with drug resistance tests, etc, they can be applied in many clinical and research laboratories in various fields. NGS platforms are used for various purposes in clinical virology laboratories Figure 1:

1. The identification of new viral pathogens

Some studies conducted for this purpose are as follows:

- The identification of viruses that cause a severe febrile illness of unknown origin (febrile hemorrhagic sepsis) by a metagenomic method [49];
- The discovery of the new Merkel Cell polyomavirus, the causative agent of Merkel cell carcinoma, etc. [50].

With the application of shotgun sequencing techniques in metagenomic studies, novel viruses can be discovered, including both pathogenic and non-pathogenic commensal viruses.

2. Ultra-deep targeted sequencing for viral community analyses

A few examples of studies conducted using this method are listed below:

- The genotyping of viruses such as HPV [51];
- The identification of HCV and HIV quasispecies [52];
- HIV, HCV, and HBV drug resistance detection, etc. [52].

3. The analysis of interactions between virus and host

The development of knowledge of the pathogenesis of viral diseases can help identify beneficial diagnostic and prognostic indicators for viral diseases, determine potential targets for antiviral drugs, and develop new vaccines. Examples of the contributions of NGS methods to virus–host interaction and pathogenesis research are as follows:

- The identification of novel viral genes and transcript isoforms during EBV and CMV infection using RNA sequencing [53,54];
- Identification of host-virus interactions in EBV infection [55].

4. Metagenomic analysis of the human virome

**Figure 2:**
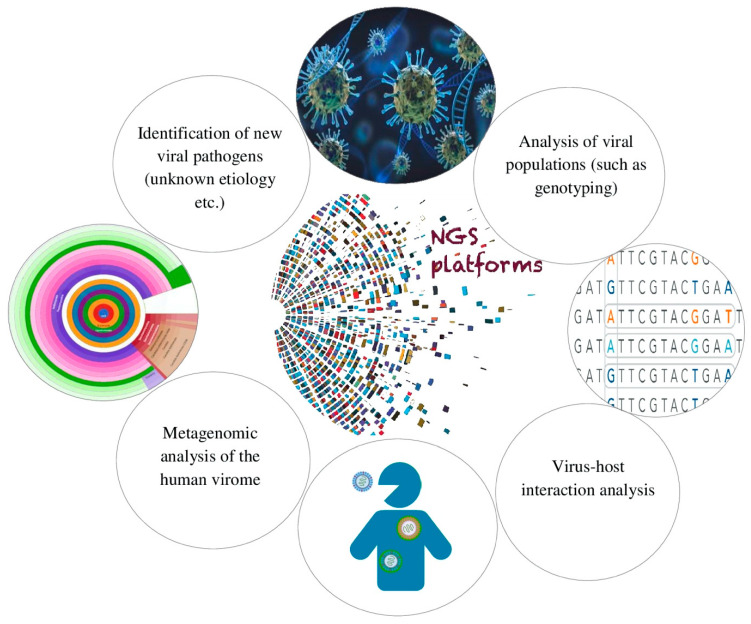
Purposes of use of NGS platforms in clinical virology laboratories.

The compounds of the human virome in both normal and illness states remain largely unexplored, although they can be deciphered using NGS methods. In this context, NGS can greatly benefit our understanding of the complex interaction between virus–host. To this end, it can assist us with the following:

- Influenza phylogenesis [56];
- The study of HIV evolution and spread [57];
- Molecular epidemiology and the surveillance of viruses such as arboviruses [58];
- The detection of mutations [59];
- The determination of suitability for antiviral treatments in the HIV and HCV genome [60];
- The monitoring of variants and viral vaccine efficacy that escape host neutralization of HIV, etc. [61] (Figure 2).

## 3. New Molecular Methods in the Diagnosis of Viral Infections

Currently, antigen-antibody-based methods and nucleic acid amplification-based methods are frequently used in the diagnosis of infectious diseases. Classical PCR methods are developed with the aim of increasing specificity and sensitivity, reducing costs, and increasing the speed of results; yet, new diagnostic methods are emerging. With the development of sequencing techniques, these methods have started to be used more in clinical microbiology laboratories. In addition to the detection of host biomarkers, omics approaches developed for the analysis of processes related to pathogen–host interactions are promising in the management of viral diseases.

Although it is said that molecular and antibody-dependent methods will dominate the diagnosis of viral diseases, the usage of these methods should be complemented by combining them with technologies such as NGS platforms [40]. NGS is used to sequence DNA and RNA nucleotide sequences and can be used to distinguish the host nucleic acid sequence from the sequences of viruses [62]. Thus, the powerful method has advantages for diagnosing viruses and will be useful in cases where the etiologic agent cannot be identified. While molecular, immune, and cell culture techniques can be used to detect previously unidentified pathogens in an investigational “elimination process” approach, NGS offers a time-efficient method to deliver the same results [63]. Therefore, while other recently used methods may be faster in diagnosing known viruses, NGS may shorten the diagnostic time for new or unexpected viral pathogens. NGS application examples, which enable the diagnosis of a previously unknown virus or identify unexpected viruses in specimens, have been reported in the literature. NGS can also be used in the emergency diagnosis of viruses and to monitor the status of specific viral diseases in a population for which diagnostic tests are not available to identify the virus [64,65,66]. In addition, in the fight against vectors for the diagnosis and control of possible new pandemics, organisms such as mosquitoes and bats can be passively sampled and tests can be always used to allow for the monitoring of virus prevalence in a host community [67,68]. 

However, while NGS is an up-and-coming technology for the diagnosis of existing viral diseases and the identification of new viruses, it entails some difficulties due to high costs, the need for specialized staff and bioinformaticians, poor sensitivity to certain infections with low viral loads, and a lack of universal sequence analysis data tools [69,70]. Therefore, NGS can work in conjunction with other existing techniques and/or new molecular methods to be developed in the future.

### 3.1. Nanopore Sequencing

Sequencing methods have some disadvantages in terms of generating short reads and being dependent on PCR. Nanopore, a simple, real-time, long-read sequencing technology that does not depend on PCR, was invented in 2014 to address these limitations [71]. The nanopore method is a third-generation sequencing technology that takes advantage of the effects of nucleotides on an electric flow to detect nucleotides as nucleic acids pass through a nano-sized protein or synthetic pore [72]. It can generate high-quality nucleotide reads longer than 10 kb without the need for chemical labeling. Additionally, full-length direct RNA sequencing can be conducted without the need for reverse transcription [73]. This method enables the detection of mutations in viral RNA and DNA. For example, SARS-CoV-2 and other respiratory viruses are multi-identified and epigenetic mutations can be detected with high sensitivity and specificity [74]. (Table 1).

### 3.2. LAMPORE Technology

This method has been used in the diagnosis of SARS-CoV-2 and combines LAMP and nanopore sequencing to identify highly scalable, multiple-gene regions. LAMP is a one-tube method used to amplify DNA and is a lower cost, faster option compared to RT-PCR. Reverse transcription loop-mediated isothermal amplification (RT-LAMP) unites the LAMP method to detect RNA using reverse transcription. The sequencing of the targeted region is amplified at a fixed heat. Characteristically, increased specificity is achieved by using four primers to amplify six different sequences in the target gene region. The number of amplified products produced in LAMP is significantly higher than in PCR-based amplification due to the use of multiple primer sets. LamPORE is based on the LAMP method to join sequencing as a procedure for high sensitivity, high specificity analysis [75]. 

### 3.3. CRISPR-Cas Technology

Clustered, regularly spaced, short palindromic repeats (CRISPR) are an immune reaction formed by bacteria to fight bacteriophages. Although the CRISPR/Cas technique has been broadly used for gene editing since 2013, its identification capability via Cas nucleases for DNA/RNA detection has recently been discovered [76]. The CRISPR effector Cas13 enzyme cleaves RNA supplemental to CRISPR RNA (crRNA) as collateral. CRISPR transducer targeting DNA endonuclease (DETECTR) is a technique that merges isothermal amplification with target-dependent Cas12a ssDNase activation. This new approach ensures attomolar sensitiveness for virus DNA discovery [77]. Specific high-sensitivity enzymatic reporter unlocking (SHERLOCK) is another technology that can separate single-nucleotide mutations with the programmable RNA targeting feature of cas13. A new DETECTR-based CRISPR/Cas12a test combining reverse transcription and loop-mediated amplification (RT-LAMP) is one of the fast identification candidates for POC tests [78]. 

In this method, the isothermal amplification of DNA and RNA in specimens is initially performed with recombinase polymerase amplification (RPA) and RT to rise the quantity of possible viral RNA targets. Then, a Cas13a enzyme with a particular guide RNA (sgRNA) for the relevant virus is attached. If the viral RNA sought is available in the sample, a signal of light that can be detected by the reader is produced as a result of binding to viral RNA via Cas13a guide RNA and releasing a fluorescently labeled reporter by collateral cleavage. In this way, the presence of the sought RNA can be determined definitively [79]. The SHERLOCK method, which is the technique developed by Gootenberg et al., can detect ZIKV and Deng virus within a few hours in patient samples (blood, urine, or saliva) where titers can be as low as 2 × 10^3^ copies/mL [80,81]. Methods such as CRISPR-based combinatorial arrayed reactions for the multiplexed evaluation of nucleic acids (CARMEN) that use microfluidic chips and thousands of targets that can be studied at the same time are also in the process of being included in these tests [82]. 

### 3.4. Microfluid Systems

Microfluidic systems are automated test devices that consist of different parts, including micro-channels that circulate liquid at the μm level, micro-pumps combined with various attachments for the analysis of samples, an inlet valve, and a reduced outlet drain [83]. These systems are a new generation of traditional detection methods consisting of steps such as sample preparation, bioreaction, and diagnosis that can be adapted to a miniature platform. In the management of viral diseases, these systems are used with high accuracy in the diagnosis of diseases such as HIV, ZIKV, and HBV [84]. The use of microfluidic chips in diagnosis significantly shortens the time between determining the etiology of the disease and initiating treatment [85]. Portable microfluidic test kits can be particularly important in areas with poor health care services. Traditional virus diagnosis methods require the isolation and purification of a large number of pathogens, among other needs associated with cell culture. The application of a small and portable diagnostic test kit significantly reduces costs as well as the length of hospital stay required for diagnosis [86]. Microfluidic systems with biodegradable materials enable tests to be carried out easily without the need for expert personnel.

Microfluidic systems can be used to detect the effects of drugs or other biochemical processes acting on cells. These microfluidic chips are grouped into those that interact with bacterial cells, biomolecules, biomarkers, and viruses [87,88,89]. Various protocols and microfluidic tests have been produced for the two- or three-dimensional detection of biological molecules. In traditional methods, such as serological tests using multi-well plates, ELISA tests, and PCR-based viral diagnostic tests, sample volumes, test times, and cost-effectiveness are not prioritized. In traditional test methods, sample collection and microorganism culture and identification are performed separately, while in microfluidic systems, all these processes can be combined into a single complex system thanks to chips [90].

## 4. Future Molecular Methods

### 4.1. Molecular Methods Based on Host Response

PCR is generally used for the detection of etiologic agents in infectious diseases. However, the PCR method has some limitations. One is that it requires a minimal pathogen load in the blood, which can, at times, lead to incorrect (or false) negative test results. Another limitation is the human bias linked to laboratory personnel performing these tests due to the repetition process of pathogen-based tests in order to increase sensitivity and specificity [91].

Host response-based immunodiagnostic techniques are a step towards more precise and personalized health care that can provide the best therapy for each patient in a well-timed approach [92]. Today, omics platforms continue to evolve around host immunodiagnostic, with different molecular host biomarkers being cited as possible candidates for a fast diagnosis in critical illnesses [93]. Unlike pathogenic-based tests, host immunodiagnostic methods offer the possibility to distinguish between non-contagious immune triggers, including sterile inflammatory cases, autoimmune illnesses, and malignity [91]. 

These methods include RT-PCR, RNA sequencing, and specific host gene expression markers. There are also platform assays to detect metabolic and protein biomarkers of susceptibility and immune response to the infectious agent. These new technologies have made it possible to join plural biomarkers into single predictive models. Therefore, recent progress has been made in epigenomics, lipidomics, and metabolomics in the combining of genomics, transcriptomics, and proteomics [94]. These approaches claim more precise identification of infectious agents, but, to date, they have not passed any known clinical research testing trials nor been approved for clinical practice.

Given the need for rapid diagnosis during the COVID-19 pandemic, several studies have discovered the usage of host-based diagnostic methods for the detection of SARS-CoV-2. One of these studies aimed to obtain a transcriptional signature to detect multiple viral infections containing COVID-19. RNA sequencing was conducted in confirmed bacterial, viral, or non-infected cases in whole blood samples from subjects. Signature host genes were confirmed with RT-PCR. IGF1R, NAGK, and HERC6 signature genes were reproduced from subjects recorded by differential gene expression analysis using forward partial-selection smallest squares. IFG1R is an insulin signal tyrosine kinase protein that has been discovered as an entry receptor for the respiratory syncytial virus (RSV) in addition to macrophage and phagocytosis activation. NAGK is one of the enzymes responsible for amino acid metabolism. HERC6 has an antiviral function when triggered by interferon. These gene transcripts distinguish bacterial infections from viral infections with 97.3% sensitivity and 100% specificity, outperforming C-reactive protein (CRP) and leukocyte counts. The three signature genes differentiated among bacterial and COVID-19 cases, with a sensitivity of 88.6% and a specificity of 94.1%, by a second confirmatory analysis [95]. 

A 29-mRNA panel—a host response-based molecular method—was developed to determine the likelihood of viral infection and the risk of physiological decompensation (i.e., disease severity) [96]. This method can be implemented on a POC platform with a turnaround period of 30 min. This method, which uses artificial intelligence algorithms, has been developed for the diagnosis and prognosis follow-up of viral diseases from whole blood. Thanks to this method in triage for suspected COVID-19 cases, it was possible to distinguish between bacterial/viral or non-infectious inflammation and to determine the course of the disease for patients in need of quarantine [97].

### 4.2. Nanobiosensors

Nanobiosensors are used in the detection of pathogens because they are cheap, sensitive, and fast and can be applied at the bedside. Biosensor technology enables target molecules to be identified and converted into electrical signals through a transducer or detector [98]. Bioreceptors targeting molecules such as nucleic acids or proteins bind to a transducer and allow the target and receptor to interact. Any interference is converted by the transducer into an electrical signal and transmitted to the detector [99]. Nanobiosensors exhibit a very high, fast, and definite target-receptor interaction [100]. The eCovSens appliance, which contains a fluorine-added tin oxide electrode and gold nanoparticles bonded with a SARS-CoV-2 antibody, can determine spike protein in saliva specimens at very low densities [101]. Nanobiosensors can be used in RT-LAMP experiments and provide synchronous amplification and complete determination in one step within one hour. Most nanobiosensors do not require the preprocessing of samples and provide a more accurate diagnosis by detecting viral particles quickly and more reliably [102]. 

Considering the use of nanobiosensors in the diagnosis of viral diseases, biosensors with highly sensitive diagnostic features have been designed to detect the influenza virus through an optical method called the resonance energy transfer upconversion luminescence process [103]. These biosensors can diagnose viruses approximately 10 times faster than traditional diagnostic methods. Today, there are studies reporting that nanobiosensors are used in the diagnosis of SARS-CoV-2 [104,105]. 

### 4.3. Omics Technologies

Omics technologies are divided into subgroups such as genomics, which allows one to examine host and microorganism genomes, transcriptomics (where transcription is examined), proteomics (where proteins are examined), and metabolomics (where metabolism products are examined). Omics research in infectious diseases can be directed towards both organisms separately for the agent and host. In addition, both can be studied under the same omics studies (such as infectomics) [106,107]. DNA sequence data and reverse genetic studies allow one to determine the effects of detected mutations on proteins. Antiviral resistance in agents such as HBV, HIV, and CMV can be detected by tests based on DNA sequence analysis [108].

A better understanding of the network structures and pathogenesis of viruses in transcriptome studies has become possible with next-generation sequencing methods [106]. The detection of minor variants that may cause drug resistance has begun to be made more sensitive with these methods. For example, in a study conducted on naïve patients infected with HIV-1, resistance-related mutations were detected in 30.5% of the samples for which in-depth sequence analysis was performed, while the rate of resistant population remained below 20% (or in 15.6% of them). This rate represents a population that can be overlooked by conventional sequencing methods [109].

Proteomic studies are in the form of general protein profiling, examining the modification forms of proteins and protein-protein and protein-genome interactions. The effects of host replication mechanisms in HIV infections and the carcinogenesis mechanisms of infections such as EBV can be better understood as a result of proteomic studies [106]. In the diagnosis of HIV-1, increases in inflammatory markers and the proteins of the complement system were detected in CSF with proteomic approaches [110]. The remodeling of cholesterol metabolism, translation, splicing, and carbon metabolism in the host was determined in the diagnosis of SARS-CoV-2 [111].

The concept of metabolomics covers the study of molecules such as amino acids, lipids, nucleotides, and sugars, which are products of metabolism. Since lipidomic studies within metabolomics provide information on molecules, including cell membrane and signaling processes, information is obtained on subjects concerning, for example, pathogen–host interactions and immune response regulation. In this way, it is possible to better understand intracellular processes in HIV, CMV, and HCV infections [106]. Another aim of metabolomic studies is to find biomarkers that can be used in rapid diagnosis by examining both the metabolic products that change in the host and the active metabolism products during infection [112].

It is thought that, thanks to the knowledge that will be obtained through future omics studies, it will be possible to take proactive early measures by observing warning signals before the symptoms of a certain health condition appear and to prevent the disease or any related sequelae. More research means more data, valuable information, and platforms for interactomics studies. Thus, in a comprehensive study on individuals with clinically slow progression in HIV-1 infected individuals, KPNA2 and ATP5G3 genes, which have key roles in nuclear transport and RNA processing, may also play an important role in terms of the disease progression. It has been reported that both these genes can be treated as key targets for new treatment methods that can be developed to slow the progression of diseases [113].

## 5. Future Directions 

Developments in metagenomic approaches in virology, the compatibility of various “omics” data with diagnostic methods, and their application, (which includes the use of teams between clinics and laboratories) require significant changes in existing health systems. These changes include the creation of special data storage and bioinformatics systems and also require the training of expert personnel in methods such as NGS. Context-aware systems using various network algorithms are online tools aimed at supporting patients and doctors in managing chronic diseases [114,115]. In the future, by using similar systems, integrating multi-omics technologies and other molecular methods in viral diseases, and developing symptom-oriented diagnosis predictions, prognosis and treatment follow-up can be realized with computer-aided applications. By integrating knowledge from advanced molecular methods and using a single mathematical model for evaluation, new intervention techniques can be used and applied to prevent and manage future epidemics.

## 6. Conclusions 

The main purpose of medical virology laboratories in the management of viral diseases is to support physicians in the diagnosis and treatment of these conditions and infection control studies. For effective disease management, causative viruses must be identified quickly. Molecular techniques are used most frequently for nucleic acid detection in the diagnosis of virus-induced infections. Molecular techniques are preferred due to their higher sensitivity and specificity, speedy results, and automation advantage compared to culture or other diagnostic methods. 

In the diagnosis of a suspected viral infection, PCR is most commonly used to confirm results obtained with antigen-/antibody-based techniques. Sequence analyses are recommended to confirm samples suspected of PCR positivity. However, sequence analyses and omics-based techniques are not practical methods that can be used in the routine clinical diagnosis of viruses due to their costs, the need for experienced personnel, and associated heavy workloads.

There is increasing evidence to suggest that the development and standardization of molecular diagnostic tests and the strengthening of the capacity to detect agents in viral diseases are of critical importance, especially after the COVID-19 pandemic. While we must develop new molecular methods to control the spread of viruses, new approaches, such as nanotechnology and artificial intelligence applications, will also allow for improvements in existing diagnostic methods. All developed molecular methods should be evaluated based on the virus to be tested. New molecular methods should be included in existing diagnostic algorithms (both national and international guidelines), taking into account parameters such as the usability, specificity, and sensitivity of these tests.

## Figures and Tables

**Table 1:** Summary of molecular techniques used in the diagnosis of viruses [9,20,21,22,23,24,25,26,27,28,29,30,31,32,33,34,35,36,37,38,39,40].

	Method	Viral Diseases	Sensitivity and Specificity of the Method
Target Amplification Techniques	PCR, RT-PCR	Many viruses RNA viruses	Sensitivity 77.8–100%, specificity 89–100% Sensitivity 73–100%, specificity 99–100%
	Nested PCR, Nested RT-PCR	Herpesviruses, HBV, EEE, WEE	Sensitivity 93.5%, specificity 100%
	LAMP	Adenovirus, CMV	Sensitivity 95.0–97.7%, specificity 92.6–99.3%
	HDA	HIV-1, HSV-1, HSV-2	Sensitivity 100%, specificity 96.3%
	NASBA	HIV, CMV, RSV	Sensitivity 90.5%, specificity 95.2–100%
Probe Amplification Techniques	LCR	HIV	Sensitivity 90–99.5%, specificity 97.6–100%
	Cyclic probe technology	VZV, HCV	Sensitivity 92.0%, specificity 75.0%
	Cleavase-invader technology	HCV, HPV	Sensitivity 96.5–98.4%, specificity 99.7%
	Hibrit capture	HBV, CMV	Sensitivity 73.7%, specificity 92.05%
	Branched DNA technique	HBV, HCV, CMV	Sensitivity 95%, specificity 95%
	Tyramide signal amplification	HPV, HSV	-
Sequencing Techniques	Sanger sequencing	Many viruses	-
	Next-generation sequencing	Many viruses	-
Other Techniques	Biosensors	HIV, HBV, Ebola virus, ZIKV, SARS-CoV-2	Sensitivity 100%, specificity 100%
	Microarray	HCV, HPV, HIV, HSV	Sensitivity 87–90%, specificity ≥99%
	Microfluid systems	HIV, ZIKV, HBV	Sensitivity 83%, specificity 100%
New Methods	Nanopore sequencing	SARS-CoV-2	Sensitivity 94.5%, specificity 91.6%
	LamPORE technology	Influenza, RSV, SARS-CoV-2	Sensitivity 99.96–100.0%, specificity 99.28–99.50%
	CRISPR-Cas technology	ZIKV, DENV, HPV	Sensitivity 88–96%, specificity 100%
	Multiplexed Microsphere-based array	HIV, HCV, HSV	Sensitivity 91.2%, specificity of 99.7%

CMV: cytomegalovirus; EEE: eastern equine encephalitis; HBV: hepatit B virüsü; HCV: hepatitis C Virus; HIV: human immunodeficiency virus; HSV: herpes simplex virus; LAMP: loop-mediated isothermal amplification; LCR: ligaz chain reaction; NASBA: nucleic acid sequence-based amplification; WEE: western equine encephalitis; PCR: polimeraz zincir reaksiyonu; RT-PCR: reverse transcriptase PCR; SARS-CoV-2: severe acute respiratory syndrome virus-2; HDA: helicase-dependent amplification; RSV: respiratory syncytial virus; VZV: varicella zoster virus; ZIKV: zika virus; DENV: dengue virus; HPV: human papillomavirus.

## Data Availability

Not applicable.

## References

[1] Hayden R., Persing D.H. (2001). Diagnostic molecular microbiology review. Curr. Clin. Top. Infect. Dis..

[2] Huizing K.M.N., Swanink C.M.A., Landstra A.M., van Zwet A.A., van Setten P.A. (2011). Rapid enterovirus molecular testing in cerebrospinal fluid reduces length of hospitalization and duration of antibiotic therapy in children with aseptic meningitis. Pediatr. Infect. Dis..

[3] Van Belkum A., Niesters H.G. (1995). Nucleic acid amplification and related techniques in microbiological diagnosis and epidemiology. Cell Mol. Biol..

[4] Ebner K., Suda M., Watzinger F., Lion T. (2005). Molecular detection and quantitative analysis of the entire spectrum of human adenoviruses by a two-reaction real-time PCR assay. J. Clin. Microbiol..

[5] Ebner K., Rauch M., Preuner S., Lion T. (2006). Typing of human adenoviruses in specimens from immunosupressed patients by PCR-fragment length analysis and real-time quantitative PCR. J. Clin. Microbiol..

[6] Ghaffari S.H., Obeidi N., Dehghan M., Alimoghaddan K., Gharehbaghian A., Ghavamzadeh A. (2008). Monitoring of cytomegalovirus reactivation in bone marrow tranplant recipients by real-time PCR. Pathol. Oncol. Res..

[7] Perandin F., Cariani E., Pollara C.P., Manca N. (2007). Comparison of commercial and in-house real-time PCR assays for quantification of Epstein-Barr virus (EBV) DNA in plasma. BMC Microbiol..

[8] Atkinson C., Emery V.C. (2011). Cytomegalovirus quantification, where to next in optimising patient management?. J. Clin. Virol..

[9] Cobo F. (2012). Application of molecular diagnostic techniques for viral testing. Open Virol. J..

[10] Almassian D.R., Cockrell L.M., Nelson W.M. (2013). Portable nucleic acid thermocyclers. Chem. Soc. Rev..

[11] Sciuto E.L., Leonardi A.A., Calabrese G., Luca G.D., Coniglio M.A., Irrera A., Conoci S. (2021). Nucleic Acids Analytical Methods for Viral Infection Diagnosis, State-of-the-Art and Future Perspectives. Biomolecules.

[12] Navarro E., Serrano-Heras G., Castaño M.J., Solera J. (2015). Real-time PCR detection chemistry. Clin. Chim. Acta.

[13] Clark K.D., Zhang C., Anderson J.L. (2016). Sample preparation for bioanalytical and pharmaceutical analysis. Anal. Chem..

[14] Fakruddin M.D. (2011). Loop mediated Isothermal Amplification (LAMP)—An Alternative to Polymerase Chain Reaction (PCR). Bangladesh Res. Pub. J..

[15] Notomi T., Okayama H., Masubuchi H., Yonekawa T., Watanabe K., Amino N., Hase T. (2000). Loop-mediated isothermal amplification of DNA. Nucleic Acids Res..

[16] Poon L.L., Leung C.S., Tashiro M., Chan K.H., Wong B.W., Yuen K.Y. (2004). Rapid detection of the severe acute respiratory syndrome (SARS) coronavirus by a loop-mediated isothermal amplification assay. Clin. Chem..

[17] Pyrc K., Milewska A., Potempa J. (2011). Development of loop-mediated isothermal amplification assay for detection of human coronavirus-NL63. J. Virol. Methods.

[18] Shirato K., Semba S., El-Kafrawy S.A., Hassan A.M., Tolah A.M., Takayama I. (2018). Development of fluorescent reverse transcription loop-mediated isothermal amplification (RT-LAMP) using quenching probes for the detection of the Middle East respiratory syndrome coronavirus. J. Virol. Methods.

[19] Kashir J., Yaqinuddin A. (2020). Loop mediated isothermal amplification (LAMP) assays as a rapid diagnostic for COVID-19. Med. Hypotheses.

[20] Saylan Y., Erdem Ö., Ünal S., Denizli A. (2019). An alternative medical diagnosis method, biosensors for virus detection. Biosensors.

[21] Chertow D.S. (2018). Next-generation diagnostics with CRISPR, CRISPR-Cas biology promises rapid.; accurate.; and portable diagnostic tools. Science.

[22] Sabalza M., Yasmin R., Barber C.A., Castro T., Malamud D., Kim B.J. (2018). Detection of zika virus using reverse-transcription LAMP coupled with reverse dot blot analysis in saliva. PLoS ONE.

[23] Gürsoy N.C., Otlu B. (2017). Mikrobiyota Çalışmalarında Moleküler Tanı Yöntemleri. J. Biotechnol. Strateg. Health Res..

[24] Hassan R., White L.R., Stefanoff C.G., de Oliveira D.E., Felisbino F.E., Klumb C.E., Bacchi C.E., Seuánez H.N., Zalcberg I.R. (2006). Epstein-Barr virus (EBV) detection and typing by PCR: A contribution to diagnostic screening of EBV-positive Burkitt’s lymphoma. Diagn. Pathol..

[25] Maignan M., Viglino D., Hablot M., Termoz Masson N., Lebeugle A., Collomb Muret R., Mabiala Makele P., Guglielmetti V., Morand P., Lupo J. (2019). Diagnostic accuracy of a rapid RT-PCR assay for point-of-care detection of influenza A/B virus at emergency department admission: A prospective evaluation during the 2017/2018 influenza season. PLoS ONE.

[26] Şimşek A., İnci A., Yıldırım A., Çiloğlu A., Bişkin Z., Düzlü Ö. (2012). Nevşehir Yöresindeki Yeni Doğan İshalli Buzağılarda Cryptosporidiosis’in Real Time PCR ve Nested PCR Yöntemleri ile Saptanması. Erciyes Üniv. Vet. Fakültesi Derg..

[27] Sadeghi Y., Kananizadeh P., Moghadam S.O., Alizadeh A., Pourmand M.R., Mohammadi N., Afshar D., Ranjbar R. (2021). The Sensitivity and Specificity of Loop-Mediated Isothermal Amplification and PCR Methods in Detection of Foodborne Microorganisms: A Systematic Review and Meta-Analysis. Iran. J. Public Health.

[28] Kim H.J., Tong Y., Tang W., Quimson L., Cope V.A., Pan X., Motre A., Kong R., Hong J., Kohn D. (2011). A rapid and simple isothermal nucleic acid amplification test for detection of herpes simplex virus types 1 and 2. J. Clin. Virol..

[29] Ayele W., Pollakis G., Abebe A., Fisseha B., Tegbaru B., Tesfaye G., Mengistu Y., Wolday D., van Gemen B., Goudsmit J. (2004). Development of a nucleic acid sequence-based amplification assay that uses gag-based molecular beacons to distinguish between human immunodeficiency virus type 1 subtype C and C’ infections in Ethiopia. J. Clin. Microbiol..

[30] Davis J.D., Riley P.K., Peters C.W. (1998). A comparison of ligase chain reaction to polymerase chain reaction in the detection of Chlamydia trachomatis endocervical infections. Infect. Dis. Obstet. Gynecol..

[31] Ihira M., Higashimoto Y., Kawamura Y., Sugata K., Ohashi M., Asano Y., Yoshikawa T. (2013). Cycling probe technology to quantify and discriminate between wild-type varicella-zoster virus and Oka vaccine strains. J. Virol. Methods.

[32] Olivier M., Chuang L.M., Chang M.S., Chen Y.T., Pei D., Ranade K., de Witte A., Allen J., Tran N., Curb D. (2002). High-throughput genotyping of single nucleotide polymorphisms using new biplex invader technology. Nucleic Acids Res..

[33] Chaudhary A.K., Pandya S., Mehrotra R., Bharti A.C., Singh M., Singh M. (2010). Comparative study between the Hybrid Capture II test and PCR based assay for the detection of human papillomavirus DNA in oral submucous fibrosis and oral squamous cell carcinoma. Virol. J..

[34] Gray E.R., Turbé V., Lawson V.E., Page R.H., Cook Z.C., Ferns R.B., Nastouli E., Pillay D., Yatsuda H., Athey D. (2018). Ultra-rapid, sensitive and specific digital diagnosis of HIV with a dual-channel SAW biosensor in a pilot clinical study. NPJ Digit. Med..

[35] Müller R., Ditzen A., Hille K., Stichling M., Ehricht R., Illmer T., Ehninger G., Rohayem J. (2009). Detection of herpesvirus and adenovirus co-infections with diagnostic DNA-microarrays. J. Virol. Methods.

[36] Tarim E.A., Karakuzu B., Oksuz C., Sarigil O., Kizilkaya M., Al-Ruweidi M.K.A.A., Yalcin H.C., Ozcivici E., Tekin H.C. (2021). Microfluidic-based virus detection methods for respiratory diseases. Emergent. Mater..

[37] Fu Y., Chen Q., Xiong M., Zhao J., Shen S., Chen L., Pan Y., Li Z., Li Y. (2022). Clinical Performance of Nanopore Targeted Sequencing for Diagnosing Infectious Diseases. Microbiol. Spectr..

[38] Ptasinska A., Whalley C., Bosworth A., Poxon C., Bryer C., Machin N., Grippon S., Wise E.L., Armson B., Howson E.L.A. (2021). Diagnostic accuracy of loop-mediated isothermal amplification coupled to nanopore sequencing (LamPORE) for the detection of SARS-CoV-2 infection at scale in symptomatic and asymptomatic populations. Clin. Microbiol. Infect..

[39] Patchsung M., Jantarug K., Pattama A., Aphicho K., Suraritdechachai S., Meesawat P., Sappakhaw K., Leelahakorn N., Ruenkam T., Wongsatit T. (2020). Clinical validation of a Cas13-based assay for the detection of SARS-CoV-2 RNA. Nat. Biomed. Eng..

[40] Reslova N., Michna V., Kasny M., Mikel P., Kralik P. (2017). xMAP Technology: Applications in Detection of Pathogens. Front. Microbiol..

[41] Paramasivam A., Priyadharsini J.V., Raghunandhakumar S. (2020). N6-Adenosine Methylation (m6A), A Promising New Molecular Target in Hypertension and Cardiovascular Diseases. Hypertens. Res..

[42] Moter A., Göbel U.B. (2000). Fluorescence in situ hybridization (FISH) for direct visualization of microorganisms. J. Microbiol. Methods.

[43] Khan M., Yoo S.J., Clijsters M., Backaert W., Vanstapel A., Speleman K., Van Gerven L. (2021). Visualizing in deceased COVID-19 patients how SARS-CoV-2 attacks the respiratory and olfactory mucosae but spares the olfactory bulb. Cell.

[44] Rensen E., Pietropaoli S., Mueller F., Weber C., Souquere S., Sommer S., Isnard P., Rabant M., Gibier J.B., Terzi F. (2022). Sensitive visualization of SARS-CoV-2 RNA with CoronaFISH. Life Sci. Alliance.

[45] Baxter A.E., Niessl J., Morou A., Kaufmann D.E. (2017). RNA flow cytometric FISH for investigations into HIV immunology.; vaccination and cure strategies. AIDS Res. Ther..

[46] Annunziato P., Lungu O., Gershon A., Silvers D.N., LaRussa P., Silverstein S.J. (1996). In situ hybridization detection of varicella zoster virus in paraffin-embedded skin biopsy samples. Clin. Diagn. Virol..

[47] Obenrader S. (2003). The Sanger Method, Dept. of Biology, Davidson College. http://www.bio.davidson.edu/Courses/Molbio/MolStudents/spring2003/Obenrader/sanger_method_page.htm.

[48] Djikeng A., Spiro D. (2009). Advancing full length genome sequencing for human RNA viral pathogens. Futur. Virol..

[49] Yozwiak N.L., Skewes-Cox P., Stenglein M.D., Balmaseda A., Harris E., DeRisi J.L. (2012). Virus identification in unknown tropical febrile illness cases using deep sequencing. PLoS Negl. Trop. Dis..

[50] Feng H., Shuda M., Chang Y., Moore P.S. (2008). Clonal integration of a polyomavirus in human Merkel cell carcinoma. Science.

[51] Barzon L., Militello V., Lavezzo E., Franchin E., Peta E., Squarzon L., Trevisan M., Pagni S., Dal Bello F., Toppo S. (2011). Human papillomavirus genotyping by 454 next generation sequencing technology. J. Clin. Virol..

[52] Barzon L., Lavezzo E., Militello V., Toppo S., Palù G. (2011). Applications of next- generation sequencing technologies to diagnostic Virology. Int. J. Mol. Sci..

[53] Concha M., Wang X., Cao S., Baddoo M., Fewell C., Lin Z., Hulme W., Hedges D., McBride J., Flemington E.K. (2012). Identification of new viral genes and transcript isoforms during Epstein-Barr virus reactivation using RNA-Seq. J. Virol..

[54] Gatherer D., Seirafian S., Cunningham C., Holton M., Dargan D.J., Baluchova K., Hector R.D., Galbraith J., Herzyk P., Wilkinson G.W. (2011). High-resolution human cytomegalovirus transcriptome. Proc. Natl. Acad. Sci. USA.

[55] Arvey A., Tempera I., Tsai K., Chen H.S., Tikhmyanova N., Klichinsky M., Leslie C., Lieberman P.M. (2012). An atlas of the Epstein-Barr virus transcriptome and epigenome reveals host-virus regulatory interactions. Cell Host Microbe.

[56] Kuroda M., Katano H., Nakajima N., Tobiume M., Ainai A., Sekizuka T., Hasegawa H., Tashiro M., Sasaki Y., Arakawa Y. (2010). Char- acterization of quasispecies of pandemic 2009 influenza A virus (A/H1N1/2009) by de novo sequencing using a next-generation DNA sequencer. PLoS ONE.

[57] Tebit D.M., Arts E.J. (2011). Tracking a century of global expansion and evolution of HIV to drive understanding and to combat disease. Lancet Infect. Dis..

[58] Baillie G.J., Galiano M., Agapow P.M., Myers R., Chiam R., Gall A., Palser A.L., Watson S.J., Hedge J., Underwood A. (2012). Evolutionary dynamics of local pandemic H1N1/2009 influenza virus lineages revealed by whole-genome analysis. J. Virol..

[59] Ninomiya M., Ueno Y., Funayama R., Nagashima T., Nishida Y., Kondo Y., Inoue J., Kakazu E., Kimura O., Nakayama K. (2012). Use of illumina deep sequencing technology to differentiate hepatitis C virus variants. J. Clin. Microbiol..

[60] Fischer W., Ganusov V.V., Giorgi E.E., Hraber P.T., Keele B.F., Leitner T., Han C.S., Gleasner C.D., Green L., Lo C.C. (2010). Transmis- sion of single HIV-1 genomes and dynamics of early immune escape revealed by ultra-deep sequencing. PLoS ONE.

[61] Cassedy A., Parle-McDermott A., O’Kennedy R. (2021). Virus Detection, A Review of the Current and Emerging Molecular and Immunological Methods. Front. Mol. Biosci..

[62] Barzon L., Lavezzo E., Costanzi G., Franchin E., Toppo S., Palù G. (2013). Next-generation sequencing technologies in diagnostic Virology. J. Clin. Virol..

[63] Adams I.P., Glover R.H., Monger W.A., Mumford R., Jackeviciene E., Navalinskiene M., Samuitiene M., Boonham N. (2009). Next-generation sequencing and metagenomic analysis, a universal diagnostic tool in plant Virology. Mol. Plant. Pathol..

[64] Naccache S.N., Peggs K.S., Mattes F.M., Phadke R., Garson J.A., Grant P., Samayoa E., Federman S., Miller S., Lunn M.P. (2015). Diagnosis of neuroinvasive astrovirus Infection in an immunocompromised adult with encephalitis by unbiased next-generation sequencing. Clin. Infect. Dis..

[65] Cordey S., Vu D.L., Schibler M., L’Huillier A.G., Brito F., Docquier M., Posfay-Barbe K.M., Petty T.J., Turin L., Zdobnov E.M. (2016). Astrovirus MLB2.; a new gastroenteric virus associated with meningitis and disseminated Infection. Emerg. Infect. Dis..

[66] Rott M.E., Kesanakurti P., Berwarth C., Rast H., Boyes I., Phelan J., Jelkmann W. (2018). Discovery of negative-sense RNA viruses in trees Infected with apple rubbery wood disease by next-generation sequencing. Plant Dis..

[67] Eiras A.E., Pires S.F., Staunton K.M., Paixão K.S., Resende M.C., Silva H.A., Ritchie S.A. (2018). A high-risk Zika and dengue transmission hub, virus detections in mosquitoes at a Brazilian university campus. Parasit. Vectors.

[68] Duarte NF H., Alencar C.H., Cavalcante KK D.S., Correia FG S., Romijn P.C., Araujo D.B., Heukelbach J. (2020). Increased detection of rabies virus in bats in Ceará State (Northeast Brazil) after implementation of a passive surveillance programme. Zoonoses Public Health.

[69] Beerenwinkel N., Günthard H.F., Roth V., Metzner K.J. (2012). Challenges and opportunities in estimating viral genetic diversity from next-generation sequencing data. Front. Microbiol..

[70] Maree H.J., Fox A., Al Rwahnih M., Boonham N., Candresse T. (2018). Application of HTS for routine plant virus diagnostics, state of the art and challenges. Front. Plant Sci..

[71] Lu H., Giordano F., Ning Z. (2016). Oxford nanopore MinION sequencing and genome assembly. Genom. Proteom. Bioinform..

[72] van Dijk E.L., Jaszczyszyn Y., Naquin D., Thermes C. (2018). The third revolution in sequencing technology. Trends Genet..

[73] Viehweger A., Krautwurst S., Lamkiewicz K., Madhugiri R., Ziebuhr J., Hölzer M., Marz M. (2019). Direct RNA nanopore sequencing of full-length coronavirus genomes provides novel insights into structural variants and enables modification analysis. Genome Res..

[74] Wang M., Fu A., Hu B., Tong Y., Liu R., Liu Z., Gu J., Xiang B., Liu J., Jiang W. (2020). Nanopore targeted sequencing for the accurate and comprehensive detection of SARS-CoV-2 and other respiratory viruses. Small.

[75] Morsli M., Anani H., Bréchard L., Delerce J., Bedotto M., Fournier P.E., Drancourt M. (2021). LamPORE SARS-CoV-2 diagnosis and genotyping, A preliminary report. J. Clin. Virol..

[76] Hou T., Zeng W., Yang M., Chen W., Ren L., Ai J., Wu J., Liao Y., Gou X., Li Y. (2020). Development and evaluation of a CRISPR-based diagnostic for **2019**-novel coronavirus. PLoS Pathog..

[77] Chen J.S., Ma E., Harrington L.B., Da Costa M., Tian X., Palefsky J.M., Doudna J.A. (2018). CRISPR-Cas12a target binding unleashes indiscriminate single-stranded DNase activity. Science.

[78] Broughton J.P., Deng X., Yu G., Fasching C.L., Singh J., Streithorst J., Granados A., Sotomayor-Gonzalez A., Zorn K., Gopez A. (2020). Rapid detection of 2019 novel coronavirus SARS-CoV-2 using a CRISPR-based DETECTR lateral flow assay. medRxiv.

[79] Freije C.A., Myhrvold C., Boehm C.K., Lin A.E., Welch N.L., Carter A., Metsky H.C., Luo C.Y., Abudayyeh O.O., Gootenberg J.S. (2019). Programmable inhibition and detection of RNA viruses using Cas13. Mol. Cell.

[80] Gootenberg J.S., Abudayyeh O.O., Lee J.W., Essletzbichler P., Dy A.J., Joung J., Verdine V., Donghia N., Daringer N.M., Freije C.A. (2017). Nucleic acid detection with CRISPR-Cas13a/C2c2. Science.

[81] Altındiş M., Feyzioğlu B. (2020). Viral Enfeksiyonlar ve SARS-CoV-2’nin Tanısında Yeni Teknolojiler. GMJ.

[82] Ackerman C.M., Myhrvold C., Thakku S.G., Freije C.A., Metsky H.C., Yang D.K., Ye S.H., Boehm C.K., Kosoko-Thoroddsen T.F., Kehe J. (2020). Massively multiplexed nucleic acid detection using Cas13. Nature.

[83] Park S., Zhang Y., Lin S., Wang T.H., Yang S. (2011). Advances in microfluidic PCR for point-of-care Infectious disease diagnostics. Biotechnol. Adv..

[84] Huang G., Huang Q., Xie L., Xiang G., Wang L., Xu H., Ma L., Luo X., Xin J., Zhou X. (2017). A rapid.; low-cost.; and microfluidic chip-based system for parallel identification of multiple pathogens related to clinical pneumonia. Sci. Rep..

[85] Chiu D.T., deMello A.J., Carlo D.D., Doyle P.S., Hansen C., Maceiczyk R.M., Wootton R.C.R. (2017). Small but perfectly formed? Successes.; challenges.; and opportunities for microfluidics in the chemical and biological Sciences. Chem.

[86] Wang Y., Yu L., Kong X., Sun L. (2017). Application of nanodiagnostics in point-of-care tests for Infectious diseases. Int. J. NanoMed..

[87] Tani H., Maehana K., Kamidate T. (2004). Chip-based bioassay using bacterial sensor strains immobilized in three-dimensional microfluidic network. Anal. Chem..

[88] Zhou J., Ren K., Zhao Y., Dai W., Wu H. (2012). Convenient formation of nanoparticle aggregates on microfluidic chips for highly sensitive SERS detection of biomolecules. Anal. BioAnal. Chem..

[89] Guan X., Zhang H.J., Bi Y.N., Zhang L., Hao D.L. (2010). Rapid detection of pathogens using antibody-coated microbeads with bioluminescence in microfluidic chips. Biomed. Microevices.

[90] Nasseri B., Soleimani N., Rabiee N., Kalbasi A., Karimi M., Hamblin M.R. (2018). Point-of-care microfluidic devices for pathogen detection. Biosens. Bioelectron..

[91] Atallah J., Mansour M.K. (2022). Implications of Using Host Response-Based Molecular Diagnostics on the Management of Bacterial and Viral Infections, A Review. Front. Med..

[92] Ramilo O., Mejias A. (2017). Host transcriptomics for diagnosis of Infectious diseases, one step closer to clinical application. Eur. Respir. J..

[93] Sweeney T.E., Khatri P. (2017). Generalizable biomarkers in critical care. Crit. Care Med..

[94] Ross M.H., Zick B.L., Tsalik E.L. (2019). Host-based diagnostics for acute respiratory infections. Clin. Ther..

[95] Li H.K., Kaforou M., Rodriguez-Manzano J., Channon-Wells S., Moniri A., Habgood-Coote D., Gupta R.K., Mills E.A., Arancon D., Lin J. (2021). Discovery and validation of a three-gene signature to distinguish COVID-19 and other viral Infections in emergency Infectious disease presentations, a case-control and observational cohort study. Lancet Microbe.

[96] He Y.D., Wohlford E.M., Uhle F., Buturovic L., Liesenfeld O., Sweeney T.E. (2021). The Optimization and Biological Significance of a 29-Host-Immune-mRNA Panel for the Diagnosis of Acute Infections and Sepsis. J. Pers. Med..

[97] Buturovic L.K.P., Antonakos N., Koufargyris P., Kontogiorgi M., Damoraki G., Cheng H., Liesenfeld O., Wacker J., Midic U., Luethy R. Comparison of a Host Response Classifier to IL-6 and Other Clinical Markers for Predicting Severe Respiratory Failure in COVID-19. Proceedings of the ECCVID—ESCMID Conference on Coronavirus Disease 2020.

[98] Misra R., Acharya S., Sushmitha N. (2021). Nanobiosensor-based diagnostic tools in viral Infections, special emphasis on COVID-19. Rev. Med. Virol..

[99] Malik P., Katyal V., Malik V., Asatkar A., Inwati G., Mukherjee T.K. (2013). Nanobiosensors, concepts and variations. ISRN Nanomater..

[100] Talebian S., Wallace G.G., Schroeder A., Stellacci F., Conde J. (2020). Nanotechnology-based disinfectants and sensors for SARS-CoV-2. Nat. Nanotechnol..

[101] Layqah L.A., Eissa S. (2019). An electrochemical immunosensor for the corona virus associated with the Middle East respiratory syndrome using an array of gold nanoparticle-modified carbon electrodes. Microchim. Acta.

[102] Zhu X., Wang X., Han L., Chen T., Wang L., Li H., Li S., He L., Fu X., Chen S. (2020). Reverse transcription loop-mediated isothermal amplification combined with nanoparticles-based biosensor for diagnosis of COVID-19. Medrxiv.

[103] Tüylek Z. (2021). Biyolojik Sistemlerde Gelecekteki Nano/Biyosensör Ürünlerine Hazırlık. Uluslararası Biyosistem Mühendisliği Derg..

[104] Krishnan S., Kumar Narasimhan A., Gangodkar D., Dhanasekaran S., Kumar Jha N., Dua K., Thakur V.K., Kumar Gupta P. (2022). Aptameric nanobiosensors for the diagnosis of COVID-19, An update. Mater. Lett..

[105] Song Y., Song J., Wei X., Huang M., Sun M., Zhu L., Lin B., Shen H., Zhu Z., Yang C. (2020). Discovery of aptamers targeting the receptor-binding domain of the SARS-CoV-2 spike glycoprotein. Anal. Chem..

[106] Fontana J.M., Alexander E., Salvatore M. (2012). Translational research in infectious disease: Current paradigms and challenges ahead. Transl. Res. J. Lab. Clin. Med..

[107] Zhang W., Li F., Nie L. (2010). Integrating multiple ‘omics’ analysis for microbial biology: Application and methodologies. Microbiology.

[108] Kuskucu M.A. (2013). Mikrobiyoloji ve İnfeksiyon Hastalıklarında Omikler ve Uygulamaya Yansımaları. ANKEM Derg..

[109] Garten R.J., Davis C.T., Russell C.A., Shu B., Lindstrom S., Balish A., Sessions W.M., Xu X., Skepner E., Deyde V. (2009). Antigenic and genetic characteristics of swine-origin 2009 A(H1N1) influenza viruses circulating in humans. Science.

[110] Guha D., Lorenz D.R., Misra V., Chettimada S., Morgello S., Gabuzda D. (2009). Proteomic analysis of cerebrospinal fluid extracellular vesicles reveals synaptic injury, inflammation, and stress response markers in HIV patients with cognitive impairment. J. Neuroinflammation.

[111] Bojkova D., Klann K., Koch B., Widera M., Krause D., Ciesek S., Cinatl J., Münch C. (2020). Proteomics of SARS-CoV-2-infected host cells reveals therapy targets. Nature.

[112] Atzei A., Atzori L., Moretti C., Barberini L., Noto A., Ottonello G., Pusceddu E., Fanos V. (2011). Metabolomics in paediatric respiratory diseases and bronchiolitis. J. Matern. Fetal Neonatal Med..

[113] Yang J., Yang Z., Lv H., Lou Y., Wang J., Wu N. (2013). Bridging HIV-1 cellular latency and clinical long-term non-progressor: An interactomic view. PLoS ONE.

[114] Klein-Geltink J., Khan S., Cascagnette P., Gershon A., To T., Crighton E. (2012). Respiratory Disease in the Métisnation of Ontario.

[115] Mcheick H., Saleh L., Ajami H., Mili H. (2017). HCES: Helper Context-Aware Engine System to Predict Relevant State of patients in COPD Domain using Naïve Bayesian. Proceedings of International Conference on Internet of Things and Machine Learning (IML 2017).

